# Intra-articular Fracture of the Distal part of the Triquetrum within the Pisotriquetral Joint: Case Report and Review of Literature

**DOI:** 10.2174/1874325001812010084

**Published:** 2018-03-16

**Authors:** V. Athanasiou, A. Panagopoulos, ID. Iliopoulos, I. Vrahnis, G. Diamantakis, P. Kraniotis, M. Tyllianakis

**Affiliations:** *Department of Hand Surgery,* Orthopaedic Clinic of Patras University Hospital, Patras, Greece

**Keywords:** Triquetrum fracture, Intra-articular fracture, Pisotriquetral joint, pPisiform subluxation, FCU subluxation, Dorsal carpal ligament tears

## Abstract

**Background:**

Intra-articular fractures of the distal part of the triquetrum within the pisotriquetral joint are uncommon, and can be associated with tears of the dorsal carpal ligaments, pisiform subluxation and/or FCU dislocation. Their diagnosis is difficult and requires a high clinical suspicion and a proper radiological examination including oblique wrist x-rays, computed tomography and MRI scan. These fractures can be delayed diagnosed due to late presentation thus leading to painful nonunion, persistent instability and late pisotriquetral arthritis.

**Case Report:**

We present a case of a 40-year-old male who complained about ulnarsided wrist pain after a fall on his extended wrist during bicycling. The diagnosis of triquetrum fracture was suspected on clinical examination and confirmed using standard and oblique radiographs and CT scan evaluation. He was immobilized in a short-arm cast for 6 weeks followed by a progressive return to wrist motion and subsequent strengthening for another 5 weeks. He reported complete resolution of pain and excellent wrist motion and function one year after the injury, demonstrating a Mayo score of 100.

**Conclusion:**

Isolated intra-articular fractures of the triquetrum within the pisotriquetral joint are rare injuries and may constitute a subcategory of body fractures other than the dorsal cortical (chip), main body and volar lip avulsion fractures. Early clinical suspicion and proper imagine can lead to a successful outcome.

## 1 INTRODUCTION

Triquetral fractures are the second most common isolated fractures of the carpal bones after scaphoid fractures [1, 2]. Three main categories have been described including *dorsal cortical chip fractures* usually after falls with the wrist in dorsiflexion and ulnar deviation; *body fractures* as a result of crushed injuries, axial dislocations, direct blow, or in the content of perilunate injuries and *volar avulsion fractures* that are considered avulsions of the palmar ulnartriquetral ligament or the lunotriquetral ligament [3-6]. Isolated intra-articular fractures of the distal ulnar part of triquetrum with or without involvement of the pisiform or pisotriquetral joint are even rarer and so far, very few cases have been reported in the literature [7-12]. These fractures can have late presentation [12] thus leading to painful nonunion, persistent instability and late pisotriquetral arthritis. We present a case of an isolated intra-articular fracture of the distal ulnar part of the triquetrum in a 40-year-old male and we provide a comprehensive review of the existing literature.

## 2 CASE REPORT

**Fig. (1):**
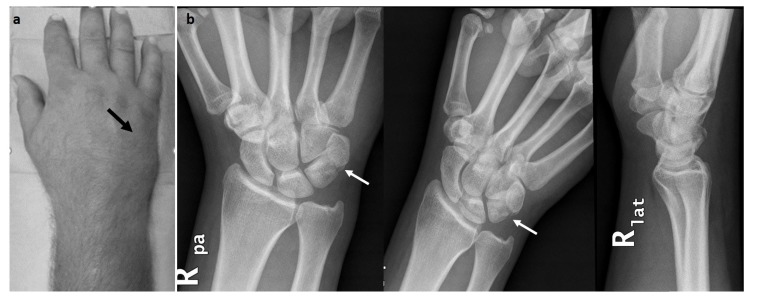
(a) Clinical presentation with marked swelling at the dorsoulnar aspect of the right wrist (black arrow), (b) posteroanterior, oblique and lateral x-rays of the wrist indicated a fracture of the triquetrum (white arrows).

**Fig. (2):**
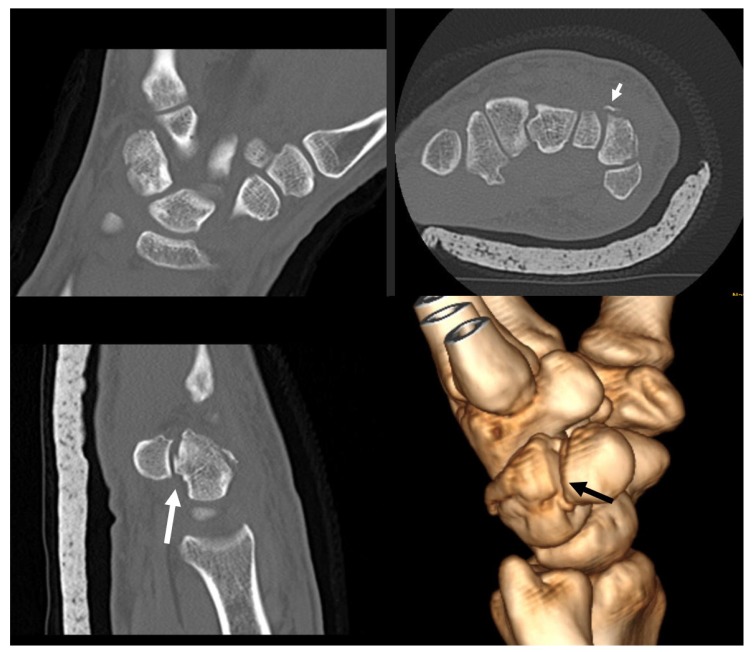
Coronal, axial and sagittal CT scan views with 3D-CT reconstruction indicated the osteochondral fracture of the distal part of the triquetrum within the pisotriquetral joint (large arrows). The small arrows indicate a tear of the dorsal intercarpal ligament (DIC).

**Fig. (3):**
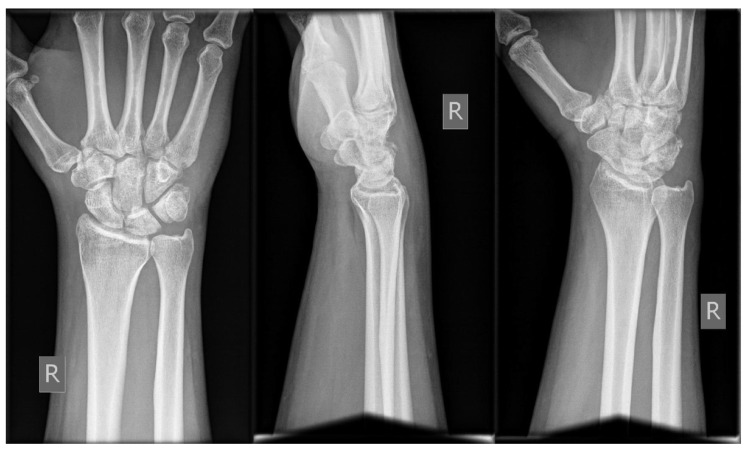
Last radiological evaluation with PA, lateral and oblique views at one year, showing healing of the fracture and normal position of the pisiform.

A 40-year-old male landed to his outstretched dominant right hand after trying to stunt on the rear wheel of his bicycle. He was seen at the hospital few hours after the injury, complaining of pain at the volar ulnar aspect of his right wrist. Physical examination revealed no pisiform instability, but a sharp pain was elicited by grinding the pisiform against the triquetrum. There was swelling at the ulnar side of the wrist and motion was limited as a result of pain (Fig. **1a**). No neurovascular compromise was detected. Ulnar deviation of the wrist exacerbated his symptoms. Radiological examination with posteroanterior, lateral and oblique views revealed a difficult to locate fracture of the triquetrum (Fig. **1b**). He had normal ulnar variance and no other abnormalities of the carpal bones in the radiographs. He underwent CT scanning of the wrist that revealed a non-displaced intra-articular fracture of the distal ulnar part of triquetrum within pisotriquetral joint and an avulsion of the dorsal intercarpal ligament (DIC) (Fig. **2**). The pisiform was not dislocated nor did it have any other pathology. He was treated in a full short arm cast for 6 weeks followed by progressive range of motion at the wrist and strengthening exercises. At his formal one-year follow up, he had a normal radiograph (Fig. **3**), full range of motion at the wrist, no pain on pisiform palpation and a Mayo wrist score of 100 points.

## 3 DISCUSSION

The triquetrum is almost a pyramid-shaped carpal bone that articulates distally with the hamate bone, proximally with the Triangular Fibro-Cartilage Complex (TFCC) without attaching the ulna and its palmar surface has an almost completely circular articulation with the pisiform [13]. The Dorsal Radiocarpal (DRC) and Dorsal Intercarpal (DIC) ligaments, forming the “diagonal V”, are the principal capsular restraints on the dorsal side of the proximal carpus [14]. The pisotriquetral joint is flat and ovoid in shape and derives its stability from the Flexor Carpi Ulnaris (FCU) tendon, the ulnar pisotriquetral ligament and the pisometacarpal and pisohamate ligaments [12].

Triquetrum fractures are usually combined with other fractures of the wrist and they are treated as part of a combined wrist injury [3, 15]. Becce *et al* [14] recently reported the MRI findings on 6 different triquetrum fracture patterns; the incidence of dorsal carpal ligament tearing was DRC: in 66,7% of cases, DIC in 76,2% of cases, and tears of the UlnoTriquetral ligaments in 81,0%. As an isolated fracture, it is the second most common carpal bone fracture after scaphoid and can also occur during athletic activities (volleyball, football, snowboarding) or bicycling as in our case [1, 7-9, 13,].

Triquetrum fractures can be divided into three types: (1) cortical dorsal or chip fractures, which are the most common; Hocker & Menschik [3] reported in 1994 a series of 231 triquetrum fractures describing the mechanism of injury (chisel action of the hamate striking against the fully extended and ulnar-deviated wrist), classification (93% chip fractures, 3% body fractures and 4% vertico-frontal fracture of the dorsal edge) and acceptable clinical results after conservative treatment with cast immobilization. (2) body fractures that can be sagittal, transverse or comminuted and often involve high-energy injuries as part of a more severe greater arc perilunate fracture-dislocation [16], and (3) avulsion fractures of the volar side of the triquetrum, first described in 1996 by Smith & Murray [6] in 5 patients as a distinct category that usually manifests a subtle sign of carpal instability; subsequent MRI examination in four wrists showed tear of the lunotriquetral ligament in each case. An additional scapholunate ligament tear was seen in one case. The avulsed volar fragment was attached to the volar scaphotriquetral ligament in one wrist, the ulnotniquetnal ligament in two wrists and the volar radiolunotriquetral ligament (VRLT) in one wrist. None of the 5 fractures was recognized on initial radiographs and all patients had persistent pain and carpal instability of variable severity at least 1 year after injury.

However, intra-articular fractures at the distal ulnar part of the triquetrum within the pisotriquetral joint with or without dislocation of the pisiform may be another rare subtype of triquetrum body fractures, although some authors utilize the term osteochondral fracture [7-9, 11]. In Table **1**, we summarize the demographic data, type of fracture, mechanism of injury and the outcome of the reported cases in the literature. In our case, this fracture was not associated with subluxation or dislocation of the pisiform.

Regarding the mechanism of injury in isolated triquetrum fractures, many theories have been proposed. The most common mechanism of injury is a fall onto an ulnarly deviated wrist in dorsiflexion. In this position, the ulnar styloid is driven as a chisel into the dorsal cortex of the triquetrum, especially when it is large in size and produces chip fractures [17]; others have suggested that the chisel action of the proximal edge of the hamate against the distal triquetrum during wrist extension is responsible for these chip fractures [3]. Becce *et al* [14] investigated also the mechanism of dorsal ligaments tearing and hypothesized a mechanism of ligament avulsion associated with specific fracture types but failed to prove different patterns between the mechanisms of avulsion and impaction. Triquetrum body fractures with a history of high energy trauma should raise the suspicion of more severe ligament injuries around the carpus, as 12% to 25% of triquetrum fractures are associated with perilunate fracture dislocations [18]. For the avulsion fractures of the volar side of the triquetrum the proposed mechanism is a moderate injury to the dorsiflexed and radially deviated wrist; in the series of Smith & Murray [6] none of the patients was diagnosed during the initial radiological screening. A common “wrist sprain” after minor trauma was evaluated later with special radiographs (in radial deviation) and the fracture was finally seen. Finally, for the intra-articular fractures the mechanism of injury is considered to be an impaction of the deviated pisiform (distally and dorsally with the wrist in hyperextension) that produce shear forces on the surface of the triquetrum. Based on this mechanism the fracture line arises from the radial to ulnar and volar to dorsal direction [9]. In our case, the mechanism of fracture was a fall onto extended wrist after a fall from bicycle.

The main clinical signs of a triquetrum fracture are tenderness to ulnar aspect of dorsal wrist, characterized as “the triquetral point” [5], swelling with or without bruising and restricted wrist motion; snapping or dislocation of the FCU tendon may be also apparent. Special clinical signs of wrist instability have to be excluded by careful examination [2]. Standard radiological workout should include posteroanterior, lateral and oblique views (15^o^ - 45^o^). However, Welling *et al*. [19] found that 20% of triquetral fractures were hidden on plain radiographs whereas Ozdemir *et al* [20] and Beccet *et al* [14] highlighted the role of oblique views plus CT and MRI scans in contrast for proper diagnosis. In our case, the standard views pointed out the presence of a triquetrum fracture and the subsequent CT scan revealed this uncommon pattern. Our patient refused to undergo a scheduled outpatient MRI scan after immobilization as he planned to travel abroad.

Treatment of triquetrum fractures depends mainly on their specific type but literature results are sparse and often obscured by the associated ligamentous injuries of the wrist. Generally, for dorsal chip fractures, especially when are accidentally identified on the lateral radiograph of an injured wrist, a short period of immobilization with a short arm cast for 3 to 6 weeks is adequate [1-3]. If pain and stiffness persist beyond 6 to 8 weeks, MRI is recommended to investigate potential ligamentous injury or TFCC tear. Dorsal chips that remain symptomatic for a long period can be excised surgically [21]. Undisplaced, body fractures, can be treated conservatively with cast immobilization [20]; in cases of marked displacement open reduction and internal fixation with a compression screw and/or Kirschner wires may be required [6, 22]. Non-union is rare but can be successfully treated with late ORIF [23]. Body fractures rarely occur in isolation and may be a radiographic indication of a higher energy and more severe wrist injury [24]. Volar triquetral avulsion fractures are considered avulsions of either the palmar ulnortriquetral ligament or the lunotriquetral ligament and usually indicate a more severe carpal instability injury that had to be thoroughly addressed [2, 6].

For the intra-articular body fractures at the distal part of the triquetrum within the pisotriquetral joint, conservative treatment in the absence of pisiform dislocation can be successfully applied as in our case. Our literature review revealed six similar cases [7-12] in young patients (5 male, 1 female, mean age 27.2 years-old; range 17 to 40) Table (**1**). Four cases described an “osteochondral” fracture and 2 a body fracture of the distal part of triquetrum located within the pisotriquetral joint. We believe that the term “osteochondral” is not appropriate as this fracture represents probably an intra-articular body fracture at the distal end of the triquetrum. Three cases were associated with athletic activities, two after falls from height and one during a motorcycle accident. The immediate diagnosis was feasible in 5 cases whereas pisiform subluxation, dislocation or instability was detected in 5/6 of the cases and determined the subsequent treatment (pisiform excision) in 3 of them. In 3 cases, the fracture was fixed by (a) closed reduction and KW, (b) arthroscopically assisted reduction and KW and (c) by open reduction and screw fixation. The clinical outcome was satisfactory in all cases after sufficient follow up period.

In general, complications of triquetral fractures can stem initially from misdiagnosis or delayed diagnosis, particularly in the presence of carpal instability. Other common causes of ulnar sided pain includes extensor carpi ulnaris (ECU) tendinitis, flexor carpi ulnaris (FCU) tendinitis, pisotriquetral arthritis, triangular fibrocartilage complex (TFCC) lesions, ulnar impaction, lunotriquetral (LT) instability, and distal radioulnar joint (DRUJ) instability [24, 25]. Jeantroux *et al* [26] highlighted also the importance of Gd-enhanced FS T1-weighted MRI sequences with wrist pronation and supination in the athletic injuries of the extensor carpi ulnaris subsheath. Nonunion or pseudarthrosis of the triquetrum is rare but can be successfully treated with fragment excision or late internal fixation [23, 27]. Painful post-traumatic pisotriquetral arthritis can also occur and can be managed with pisiform excision [9, 28].

## CONCLUSION

Inta-articular body fractures of the distal part of the triquetrum within the pisotriquetral joint are rare injuries and may be constitute a subcategory of body fractures other than the dorsal cortical (chip), main body and volar lip avulsion fractures. When a traumatic event at the volar aspect of the extended and ulnarly deviated wrist is associated with pain exacerbated by grinding the pisiform against the triquetrum an oblique x-ray of the wrist followed by CT and MRI scan can reveal the unusual pattern of the fracture and the possibility of dorsal carpal ligamentous tearing. If the fracture is non-displaced and the pisotriquetral joint is intact, conservative treatment with cast immobilization is recommended; in displaced fractures or at late presentation ORIF with or without pisiform excision is the preferred method of treatment. In any case, careful clinical examination and proper imaging have to rule out associated complex types of wrist instability.

## Figures and Tables

**Table 1:** Review of literature of isolated intra-articular fractures of the distal ulnar part of triquetrum.

**Author, year**	**Fracture type, Symptoms**	**Age, Gender**	**Mechanism of injury**	**Treatment**	**Results**
*Pevny *et al*^7^, 1996*	Osteochondral Chronic pain and recurrent radial dislocation of the pisiform	17, male	Fall on outstretched arm during volleyball	Pisiform and osteochondral fragment excision	Satisfactory
*Maeda *et al*^8^, 1998*	Osteochondral Chronic pain and recurrent radial dislocation of the pisiform	17, male	Fall on outstretched arm during football	Pisiform and osteochondral fragment excision	Satisfactory
*Suzuki *et al*^9^, 2002*	Osteochondral Pisiform instability	21, female	Fall on outstretched hand while snowboarding	Pisiform excision	Satisfactory
*Hsieh *et al*^10^, 2009*	Body fracture (distal part)	29, male	Motorcycle accident; collision with a panel truck	Closed KW fixation	Satisfactory
*Kanaya *et al*^11^, 2013*	Osteochondral Pisiform instability, LTQ ligament instability	40, male	Fall from 4 m height	Arthroscopically assisted KW fixation	Satisfactory
*Gan *et al*^12^, 2015*	Body fracture (distal part) Pisiform subluxation	29, male	Fall from height and landed on his right hand, presented 2 months later	Screw fixation	Satisfactory
